# Elements of Latin

**Published:** 1898-07

**Authors:** 


Elements of Latin. For Students of Medicine and Pharmacy. By George D. Crothers, A.M., M.D., Teacher of Latin and Greek in the St. Joseph (Mo.) High School; formerly Professor of Latin and Greek in the University of Omaha; and Hiram H. Bice, A.M., Instructor in Latin and Greek in the Boys' High School of New York City. 5¼ x 7½ inches. Pages xii–242. The F. A. Davis Co., Publishers, 1914–16 Cherry Street, Philadelphia; 117 W. Forty-second Street, New York City; 9 Lakeside Building, 218–220 S. Clark Street, Chicago, Ill.

While this book is not intended as a means for the requirement of Latin in its general sense, it certainly does give a clear conception of the use of this language as applied to medicine. The nomenclature of medicine is a wordy labyrinth that the student, even with some knowledge of Latin and Greek, finds himself in a maze of difficulties. It is in fact a new language to him, which must be learned with all the usual labor given to the acquirement of words. This is more readily accomplished in proportion to the amount possessed of the original tongues from which most of the words used in medicine or dentistry are derived or formed. It is, therefore, a great saving of time to have a work of authority as complete as this to refer to as occasion demands.

Some students enter upon the study of medicine who have taken their preliminary training in schools where Latin is not made an obligatory study. These, fortunately, are comparatively few, but for these this book is especially recommended. In fact, it is difficult to understand how the study of medical and dental terms can be accomplished by these without the aid of this or a similar book. To familiarize one's self with the roots of words is more than half the battle in the struggle with definitions.

